# Supplementary material for: Merging conformational landscapes in a single consensus space with FlexConsensus algorithm
Source: Nat Methods. 2025 Sep 25;22(10):2118–26. doi: 10.1038/s41592-025-02841-w (PMC12510870; doi:10.1038/s41592-025-02841-w)
Supplement: Supplementary file 1 — Supplementary information file, Supplementary figures and Supplementary videos. [file 41592_2025_2841_MOESM1_ESM.pdf]

nature methods

Supplementary information https://doi.org/10.1038/s41592-025-02841-w

Merging conformational landscapes in a single consensus space with FlexConsensus algorithm

In the format provided by the authors and unedited

Contents

Supplementary results **2**
Simulated conformational landscape with different dimensions 2
Simulated conformational landscapes with variable stability 4
Consensus results on synthetic spaces with different intrinsic dimensions 6
FlexConsensus consistency analysis 9

Supplementary methods **9**
Normalizing FlexConsensus errors 9

Supplementary results

Simulated conformational landscape with different dimensions

One of the first issues when comparing different heterogeneity algorithms is the variable nature of the conformational landscapes they generate. Apart from the intrinsic differences related to the assumptions that they consider, a mismatch may also exist in their dimensions. The dimensional differences make the consensus evaluation challenging and should be addressed in the first place to produce meaningful and interpretable comparison metrics.

In FlexConsensus, we propose a multi-encoder architecture relying on a common latent space to overcome the previous issue, as further described in the *Methods* section. The proposed architecture effectively reduces the multidimensional conformational landscapes of different methods to a new latent space with unified dimensions, thus simplifying their comparison. Assuming that the number of input cryoEM images is N and the number of different input landscapes is M, the number of points in the common latent space will be $N \times M$, and we will always know from which of the input landscapes a particular point in the common latent space is coming from. Note that each of the conformational spaces input to FlexConsensus may be a latent space in its own right (because it may come from some form of auto-encoder architecture, for example); however, we will always refer to them as input spaces, keeping the reference to latent space to the new and common space obtained by FlexConsensus.

To determine the performance of our multi-encoder architecture under the previously described conditions, we designed a simple synthetic case with two different input landscapes. We will refer to "Consensus space 1" and "Consensus space 2" as the mapping into the new common latent space of the points coming from the first and second input spaces, respectively. It should be noted that FlexConsensus does not restrict the number of conformational landscapes to be analyzed, and the choice of two input spaces is only for simplicity in the presentation.

The synthetic landscapes were generated using sine and cosine functions for the even and odd dimensions of the target space. The generation process is thus similar to the definition of the well-known Lissajous curves, but applied to the multidimensional case. This process allows the generation of spaces with similar characteristics and varying dimensions. We can express the equation of the curves as:

$$f(t) = (\sin(t), \cos(2t), \sin(3t), \cos(4t), \sin(5t), ...) \quad (1)$$

where t takes values in $[0, 2\pi]$.

For the current tests, we generated two input spaces of 1000 points in the range $[0, 2\pi]$, each with 8 and 39 dimensions, respectively, replicating the default number of dimensions of two entirely different heterogeneity algorithms in the field: CryoDRGN and Zernike3D.

We want to highlight that the proposed test represents a simplified case for evaluating and understanding the method's performance, as the input spaces have an intrinsic dimension equal to one, even if they are constructed to have a larger number of dimensions.

The results obtained using FlexConsensus with the previous synthetic spaces are summarized in Supplementary Figure 1. The training workflow starts with the input spaces being forwarded through their encoding network (as further detailed in the Methods section), producing a consensus latent space composed of "Consensus space 1" and "Consensus space 2". The consensus space is then forwarded through the decoders responsible for recovering the original input spaces from the consensus latent space. At this point, we can measure a representation loss function between the decoded and the input spaces, which will be used by the network in conjunction with other losses during the training phase.

The differences in the dimensions between the input spaces make it hard to determine whether the network has produced an appropriate consensus space. Therefore, we proposed two ways to evaluate the network's accuracy: analyzing 1) the final representation loss computed as the distance between the input and decoded spaces and 2) the Earth Mover's Distance (EMD) between the distance distributions among different spaces. Distances effectively capture the spatial characteristics of a given space, making the EMD an excellent candidate for determining the accuracy of the network predictions independently of their initial dimension.

The decision behind choosing EMD over other distance measurements, such as the L^2 norm between the spaces, is due to the possibility of computing EMD independently of the initial dimension of the spaces being compared. To achieve this, the all-to-all distances of a given space against itself are computed and used to get the distance distribution of the points composing the space. The obtained distance distributions are then used as the input for the EMD distance to measure the similarity between the input spaces. By measuring the similarities at the level of distributions, it is possible to compare the initial spaces even if they have different dimensions.

The analysis starts by measuring the EMD between the input spaces to have a reference value to compare the following EMD metrics to be measured. We will use this reference value to normalize the computed EMD values. We will refer to this metric as EMD_p. Even if the input spaces have similar shapes by construction, the EMD results in a large value, mainly due to differences in the dimensions of the inputs.

The next step consists of encoding the input spaces into the common latent space, leading to "Consensus space 1" and "Consensus space 2" as shown in Supplementary Figure 1. Similar to the case before, the EMD between the "Consensus space 1" and "Consensus space 2" was measured, yielding a small value compared to the reference EMD computed before. Both the EMD_p and the visual inspection of the landscapes effectively show that FlexConsensus has correctly determined that the input spaces were very similar, as expected (note that the curves of red and blue points in the consensus landscape map are virtually one on top of the other).

Lastly, "Consensus space 1" and "Consensus space 2" were forwarded through the different decoders, generating four decoded spaces. Two of these spaces correspond to the transformation of "Consensus space 1" and "Consensus space 2" into the first input space, while the other two come from the transformation of "Consensus space 1" and "Consensus space 2" into the second input space. At this point, we proposed two evaluation metrics: the first one is the EMD_p between the input spaces and their analogous decoded spaces, yielding a total of 4 measurements. Again, these metrics show a small value as FlexConsensus has correctly learned to accurately reproduce the input spaces from the consensus latent space.

The second evaluation is obtained from the pairwise comparison of the input spaces and their analogous decoded spaces (corresponding to the representation loss used to train the neural network), represented as a histogram to simplify their understanding. The errors presented in all the histograms shown along the manuscript were normalized to be in the range $[0, 1]$ according to the following formula:

$$f(x) = \frac{1}{1 + e^{-\frac{ln(3)}{\sigma}k(x-(\mu+\sigma))}} \tag{2}$$

where μ and σ represent the computed errors' mean and standard deviation. When this transformation is applied, the errors will follow the next constraints:

$$\begin{aligned} f(\mu) &\approx 0.25 \\ f(\mu+\sigma) &\approx 0.5 \\ f(\mu+2\sigma) &\approx 0.75 \end{aligned}$$

A detailed description of the characteristics of this transformation is provided later in this Supplementary Information file.

In agreement with the EMD_p metrics, the histograms presented in Supplementary Figure 1 show that the representation error is small (most of the errors are below $f(\mu+\sigma)$, being $\sigma = 0.23$), meaning that FlexConsensus has correctly learned to decode the input spaces from the consensus.

Simulated conformational landscapes with variable stability

Although the dataset analyzed in the previous section helps to evaluate the method's performance when faced with multidimensional data sets, it fails to reproduce the errors that disrupt the conformational landscapes in a real-case scenario. Among the errors that affect the estimation of conformational landscapes, the noise contaminating the experimental cryoEM images is the most predominant. This noise is transmitted to conformational landscape estimations, which is one of the reasons why consensus among several estimations is desired to improve the reliability and interpretation of conformational landscapes.

Therefore, a new test case is proposed to determine FlexConsensus's ability to discern which regions of the input spaces can be trusted and which areas are more questionable due to estimation differences (or, at least, deserve special attention since they are highlighted only by a subset of methods). Similarly to the previous experiment, we generated two input spaces following the Lissajous-like construction method proposed earlier. However, in this case, constructing the input spaces includes some additional steps to simulate some of the effects experimental latent spaces may have.

Focusing on the first input space, the construction begins by generating 1000 8D points, as described in the previous section. These 1000 points are then duplicated, followed by a translation of the duplicated points so they do not overlap with the original ones. This way, we obtained two replicas of the same 8D space at distinct locations. Lastly, Gaussian noise was applied to the translated replica, leading to the final input space consisting of one structured region following the Lissajous curves (the original 1000 points) and another region that did not show any apparent structure due to the added high noise (the noisy duplicated 1000 points). The standard deviation of the noise added to the points was large enough to disrupt their structure completely ($\sigma = 10$).

The second input space follows the same construction process as the previous one, starting with 1000 39D points. After generating the noiseless and noisy translated replica with 1000 39D points each, the 2000 points were rotated 90 degrees around the z-axis, crossing their center of mass. This rotation simulates the randomness in the orientation of experimental spaces, as they could be randomly rotated even if the method responsible for generating that space is executed twice with the same parameters.

The new input spaces (two input spaces, each with 1000 noise-free points and 1000 noisy data points) were fed to FlexConsenus to train the neural network. This allowed the method to learn the common consensus space and directly decode the input spaces to evaluate the representation losses. Note that we will have 2×2000 points in the consensus latent space, 2000 from each input space. However, and only for the sake of simplicity in the presentation, we will split the analysis of FlexConsensus into two sections. In the first section, we will address the performance holding the two noise-free spaces (Supplementary Figure 2a), and in the second section, we will study the results with the noisy spaces (Supplementary Figure 2b). Similarly to the layout followed in Supplementary Figure 1, the analysis starts by measuring the EMD between the points in the input spaces to get a reference value to compare. It follows with a visual inspection of the consensus latent space, and it ends with the presentation of error metrics in the form of histograms (see Methods section) and the EMD between the two decoded spaces and the input spaces. In addition, we will normalize the EMD values by computing the EMD between the noiseless points to get EMD_p.

Regarding the 2×1000 noiseless structured points analysis in the input spaces, results are summarized in Supplementary Figure 2a. As expected, the EMD value between input spaces was the same as the one measured in the previous section, as the noiseless points were constructed following the same process in

both experiments. As for the evaluation of the noiseless "Consensus space 1" and "Consensus space 2" (blue and red points in Supplementary Figure 2a), visual inspection shows that the two sets of 1000 points map virtually map one on top of each other; note that in this consensus space, the 2×2000 points coming from the input spaces are shown, although only the 2×1000 points corresponding to the noiseless input spaces are colored (the additional 2×1000 points coming from the noisy spaces are kept in gray and will be analyzed in the second section). Lastly, "Consensus space 1" and "Consensus space 2" were fed to the decoders to generate the four decoded spaces, similarly to the process followed with the first test case. Therefore, it is possible to measure the EMD between the two decoded spaces analogous to the first input space, the EMD between the two decoded spaces analogous to the second input space, and the histograms computed from the representation errors arising from the pairwise comparison of the input and the decoded spaces. As done with the consensus spaces, the error histograms computed from the noiseless subset of points are highlighted in red and blue. We kept the errors arising from the noisy points in the histograms in gray to simplify their comparison. As can be seen from the histograms and the small EMD_p distances, FlexConsenus has effectively learned to reproduce the noiseless points in the input spaces from the noiseless points in the common consensus space.

The second section analyzes the two noisy input spaces with 1000 points, as shown in Supplementary Figure 2b (blue and red points). Again, we start by measuring the EMD between the noisy points in the input space, leading to a larger reference value due to the addition of Gaussian noise. The analysis by visual inspection of the noisy "Consensus space 1" and "Consensus space 2" shows two clouds of points without apparent structure or overlap. Additionally, the EMD_p measured between them was larger, indicating that FlexConsensus fails to identify any similarity between these points. This is expected, as FlexConsensus should be able to determine that the two sets of very noisy points in the two input spaces are no longer similar due to the noise. Lastly, the analysis of the four spaces decoded from the noisy "Consensus space 1" and "Consensus space 2" show a similar trend, leading to larger errors in both the EMD_p measurements and the histograms obtained from the representation errors coming from the comparison with the 1000 noisy points in the input spaces.

The previous experiment shows that FlexConsensus is sensitive enough to correctly determine which regions in different latent spaces are more or less reliable, even if the original spaces have different characteristics, orientations, or dimensions.

Consensus results on synthetic spaces with different intrinsic dimensions

The dataset previously analyzed represents a simplified illustrative case in which the intrinsic dimensionality of the spaces was very low (just 1), even if the samples were forced to live in spaces with different dimensions. Consequently, although the previous setup helps conceptualize the method, it fails to capture the

complex relationships that may be present in real scenarios where the intrinsic dimensionality of each input space is unknown and might be different for each method.

Therefore, we conducted an additional experiment on a new synthetic dataset that, by construction, had input spaces of different intrinsic dimensionality. In addition, we imposed several non-linear constraints to better evaluate the performance of FlexConsensus on a more realistic but still controlled scenario.

The new dataset comprises two different spaces, the first based on a helix curve:

$$f(t) = (\cos(t), \sin(t), t, \sin(4t), \sin(5t), ...) \tag{3}$$

Where t takes values in $[0, 2c\pi]$, the constant c allows the control of the total number of turns of the helix. In addition, half of the turns of the previous space were further modified to introduce a non-linear transformation that helps evaluate whether FlexConsensus can detect this dissimilarity. The proposed non-linear transformation consists of a non-linear deformation that stretches the helix from its middle turn onward, whose strength depends on the distance to the center of the helix. In addition to this non-linear deformation, noise is added to these stretched regions. The noise added follows a normal distribution whose standard deviation depends on the distance to the center of the helix:

$$f(t) = \begin{cases} (\sin(t),\ \cos(t),\ t,\ \sin(4t),\ \dots), & t \leq c\pi \\ \begin{pmatrix} \sin(t) + \sigma(r),\ \cos(t) + \sigma(r), \\ t + \sigma(r),\ \sin(4t)\cos(5t) + \sigma(r),\ \dots \end{pmatrix}, & t > c\pi \end{cases} \tag{4}$$

Being $\sigma(r)$, the deformation and noise added to the original space, depending on the distance to the center of the helix r.

The second space is based on a helix-shaped surface defined by:

$$f(t) = (a\cos(t), a\sin(t), t, \sin(4t)\cos(5t), \sin(5t)\cos 6t, ...) \tag{5}$$

Where t takes values in $[0, 2c\pi]$, and a is a random number taken from a uniform distribution. Similarly to the case before, the constant c allows the control of the total number of turns of the helix.

As can be seen from the equations of the new spaces, apart from having an intrinsically different dimensionality, they have been constructed without coincident dimensions, trying to make them more challenging. The two generated spaces consist of 5000 points each and have 20 and 40 dimensions, respectively, and eight turns. After analyzing these spaces with FlexConsensus, the results are summarized in Supplementary Figure 3.

On the left-hand side of the Figure, we present a subregion of the two original spaces around the middle point of each space. To simplify their visualization, the first three dimensions of each space were aligned and scaled so they could be more easily compared. It should be noted that the spaces input to the network were not aligned or scaled by any means.

Regarding the consensus space learned by the network, the central image from Supplementary Figure 3 shows that FlexConsensus has correctly identified

that the initial turns from each space are similar. In contrast, the last turns are assigned a more significant consensus error, as expected. This result shows the ability of the method to identify the similarities and differences between the input spaces, even if they have a different intrinsic number of dimensions. Interestingly, the consensus space also shows that FlexConsensus neglects the non-linear deformation added to the final half of the turns. It correctly identifies that it should "stretch" those turns in the helix-shaped surface to match the helix curve samples. This result confirms that FlexConsenus should not be affected by non-linear modifications of the input spaces as long as the position of the samples in these spaces is consistent.

Complementing the previous results, we present the isolated consensus space for each input on the right-hand side of the Figure, highlighting in yellow those points identified to have a significant consensus error. As expected, the samples identified to have a larger consensus error are located in the half turns that were more different by construction (i.e., the region modified with noise and a non-linear deformation in the helix curve dataset). Therefore, it is possible to conclude that FlexConsensus can correctly estimate which regions in the input spaces are compatible or incompatible.

Comparison of FlexConsensus against popular dimensionality reduction methods

Although the primary purpose of FlexConsensus is to find the mapping that allows merging of different conformational spaces into a common consensus space, it also reduces the dimensionality of the input data based on its different cost functions and regularizations. Therefore, it is interesting to assess how the dimensionality reduction capabilities of FlexConsensus compare to other standardized methods such as t-SNE or distance-based clustering.

To simplify the comparison, we decided to compare the FlexConsenus spaces obtained from HetSIREN for the EMPIAR-10028 and SARS-CoV-2 datasets presented in the manuscript against the dimensionality-reduced version yielded by t-SNE and distance-based clustering from the original HetSIREN latent space. The previous comparison is summarized in Supplementary Figure 4. As can be seen from the comparison, FlexConsensus and t-SNE tend to reproduce the different local features of the landscape better than distance-based clustering.

For the EMPIAR-10028 dataset, both FlexConsenus and t-SNE properly capture the three main regions detected by HetSIREN, corresponding to the three main conformational states it detects. In contrast, distance-based clustering only detects two clouds, as highlighted in the image with the dotted ellipses.

In the SARS-CoV-2 landscape, the differences between FlexConsensus, t-SNE, and distance-based clustering are more apparent. The first two are the only ones able to detect the three main states found by HetSIREN properly.

FlexConsensus consistency analysis

Since FlexConsensus follows a neural network approximation, every time it is executed, it will produce a different estimate of the consensus space due to the stochastic nature of the neural network weights' optimization process. Although this will be true for all the executions, it would be desirable that the minima found by the network yield a consensus space with similar characteristics in all the executions to ensure that the network results are reproducible.

To better assess the reproducibility of the network, we conducted a test to evaluate how similar the consensus spaces predicted after training several FlexConsensus networks on the same dataset are. Following the previous idea, we trained five different consensus networks using the previously presented helix-based synthetic dataset. The overall consensus spaces predicted by the networks (i.e., the space obtained by joining the consensus space associated with each input) were then compared using the information imbalance metric:

$$\Delta(d_A \rightarrow d_B; k) = \Delta_{AB} = \frac{2}{N^2 k} \sum_{\substack{i,j \\ s.t r_{ij}^A < k}} r_{ij}^B \tag{6}$$

The results obtained are summarized in Supplementary Figure 5. As can be seen from the information imbalance plots, all the executions of FlexConsensus yield an information imbalance value close to zero and to the main diagonal, which verifies that the executions have yielded consensus spaces containing similar information in all cases.

Supplementary methods

Normalizing FlexConsensus errors

One of the main issues when working with errors and error histograms is determining whether a given error is large or small compared to the rest. This issue prevents us from easily determining which threshold should be set for the consensus to extract only those particles that reliably fulfill a given criterion.

Therefore, we propose a transformation based on a sigmoid function with additional conditions imposed to simplify the comparison and understanding of the error metrics. These three conditions are:

$$\begin{aligned} f(\mu) &\approx 0.25 \\ f(\mu + \sigma) &\approx 0.5 \\ f(\mu + 2\sigma) &\approx 0.75 \end{aligned}$$

Where μ and σ are the computed errors' mean and standard deviation, respectively. The second condition can be easily achieved by shifting the sigmoid function such that:

$$f(x) = \frac{1}{1 + e^{-k(x-(\mu+\sigma))}} \tag{7}$$

Fulfilling the other two conditions requires finding an appropriate slope value for the sigmoid function k. If we focus on the first condition and solve for the value of k, it is possible to get:

$$k = \frac{ln(3)}{\sigma} \tag{8}$$

If we plug the previous value into the sigmoid equation, it is possible to check that it verifies the third condition. Thus, the final transformation reduces to:

$$f(x) = \frac{1}{1 + e^{-\frac{ln(3)}{\sigma}k(x-(\mu+\sigma))}} \tag{9}$$

This transformation allows the mapping of the original error values to the interval [0,1] while being robust to outliers and providing a sense of which errors are large or small by comparing them to the standard deviation of the original data.

We would like to comment on the slope constraints selected for the sigmoid function. While the natural choice would have been to set $f(\mu) \approx 0.5$ instead of $f(\mu) \approx 0.25$, we have preferred the latter to give more resolution to the largest errors. In this way, it should be easier to set a threshold to filter high-consensus errors more accurately.

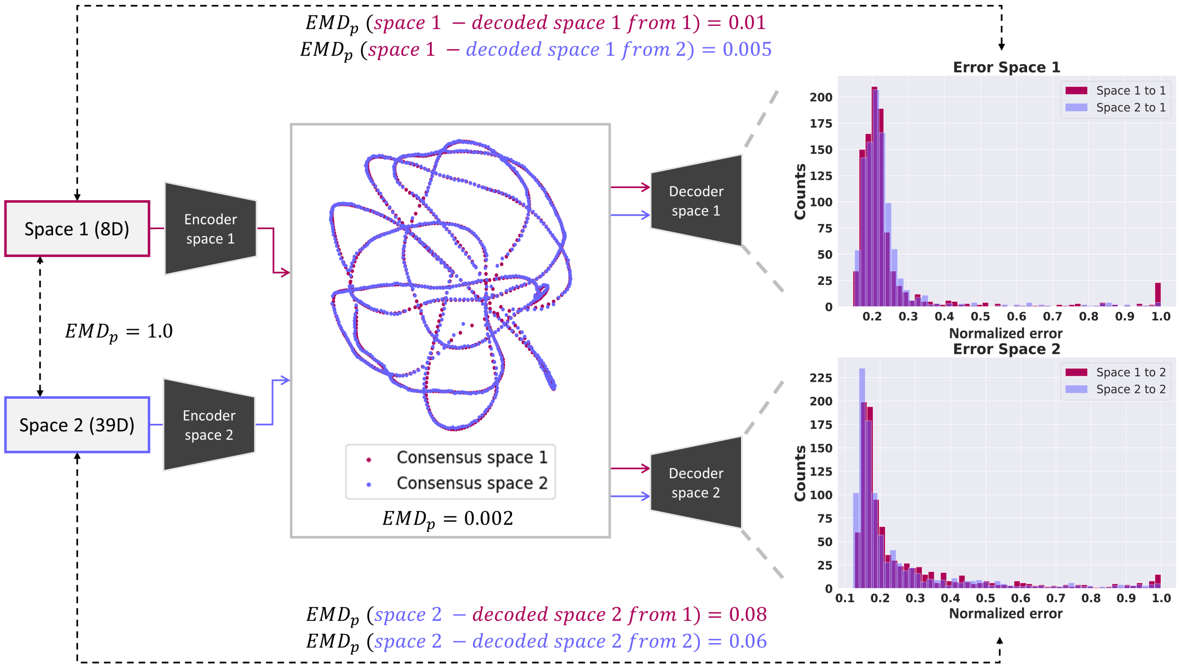

Supplementary Figure 1: FlexConsensus analysis resulting from the comparison of two synthetic spaces with a varying number of dimensions. The resulting consensus space, error histograms derived from the representation error computed by comparing the decoded and input spaces, and the EMD_p evaluation metrics estimated from the analysis are also presented in the Figure. The analysis shows that FlexConsensus identified that the two input spaces have similar characteristics, which is to be expected by construction. Additionally, the analysis of the representation error computed between the input and the decoded spaces is low. These results show the accuracy of the method even when faced with multidimensional datasets.

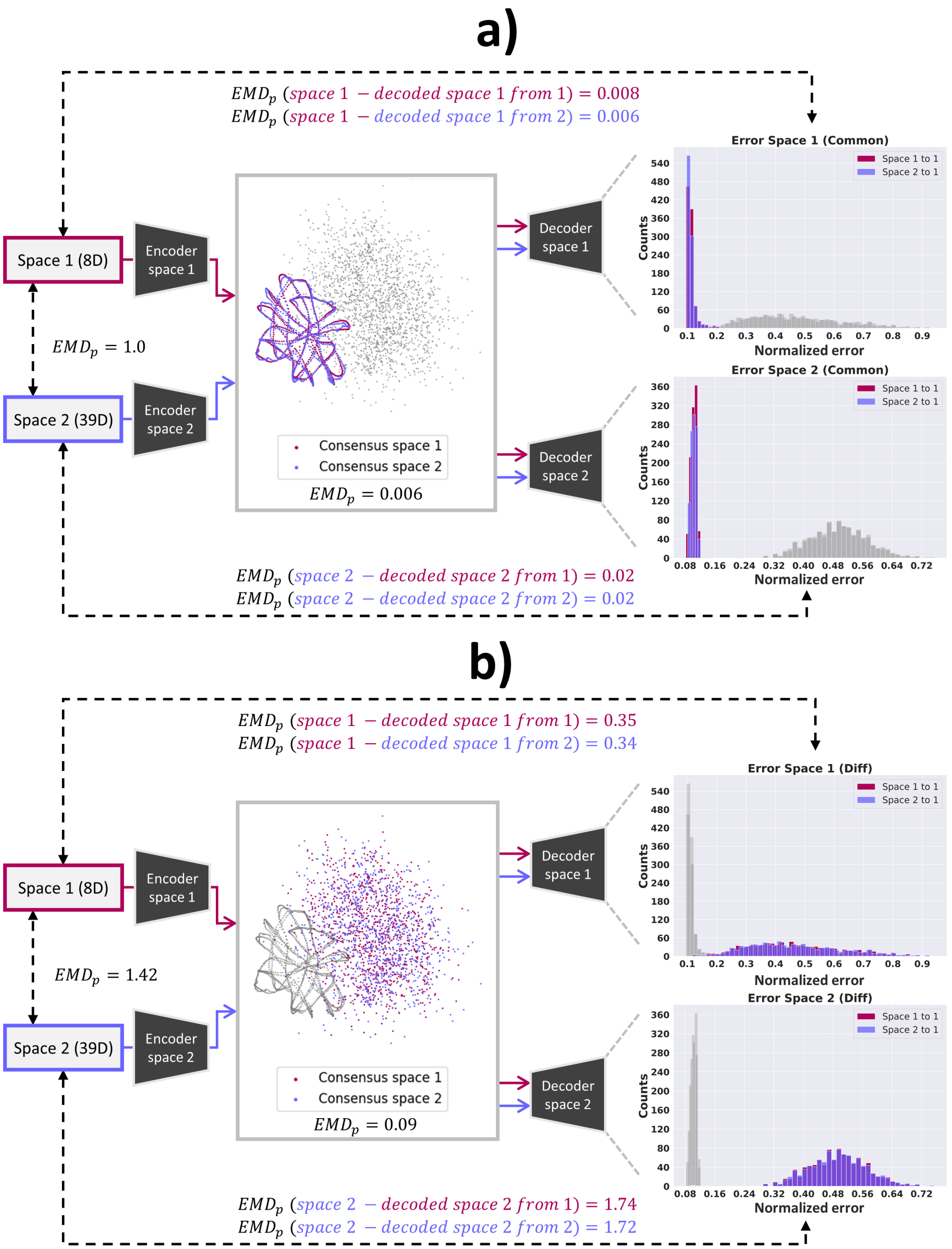

Supplementary Figure 2: FlexConsensus analysis resulting from the comparison of two synthetic spaces with a varying number of dimensions. In addition, the synthetic spaces also mimic the presence of noise and other errors found in typical experimental latent spaces. Panel a) compares the consensus space corresponding to the 2×1000 points in the input spaces with no noise added to them. It should be noted that the consensus space was computed from the full set of 2×2000 points, including the noiseless and noisy samples. However, in this first Panel, the analysis focuses only on the noiseless points highlighted in red and blue (the additional 2×1000 points coming from the noisy spaces are kept in gray and will be analyzed in the next Panel). The resulting consensus landscape, error histograms derived from the representation error computed by comparing the decoded and input spaces, and the EMD_p evaluation metrics estimated from the analysis are also presented in the Figure. As done previously in the consensus spaces, the normalized error histograms highlight the errors computed from the noiseless points in red and blue. The errors associated with the noisy points are highlighted in gray and will be analyzed in the following Panel. These results show that FlexConsensus successfully identified these regions with similar structural properties. In contrast, Panel b) focuses the analysis on the 2×1000 translated points affected by noise in the input spaces. Similarly to the case before, the analysis focuses on the noisy samples highlighted in red and blue. However, the consensus space was computed from the complete set of points, including those colored in gray. The analysis shows that FlexConsensus identifies these two regions as unreliably estimated, as they are heavily affected by the noise.

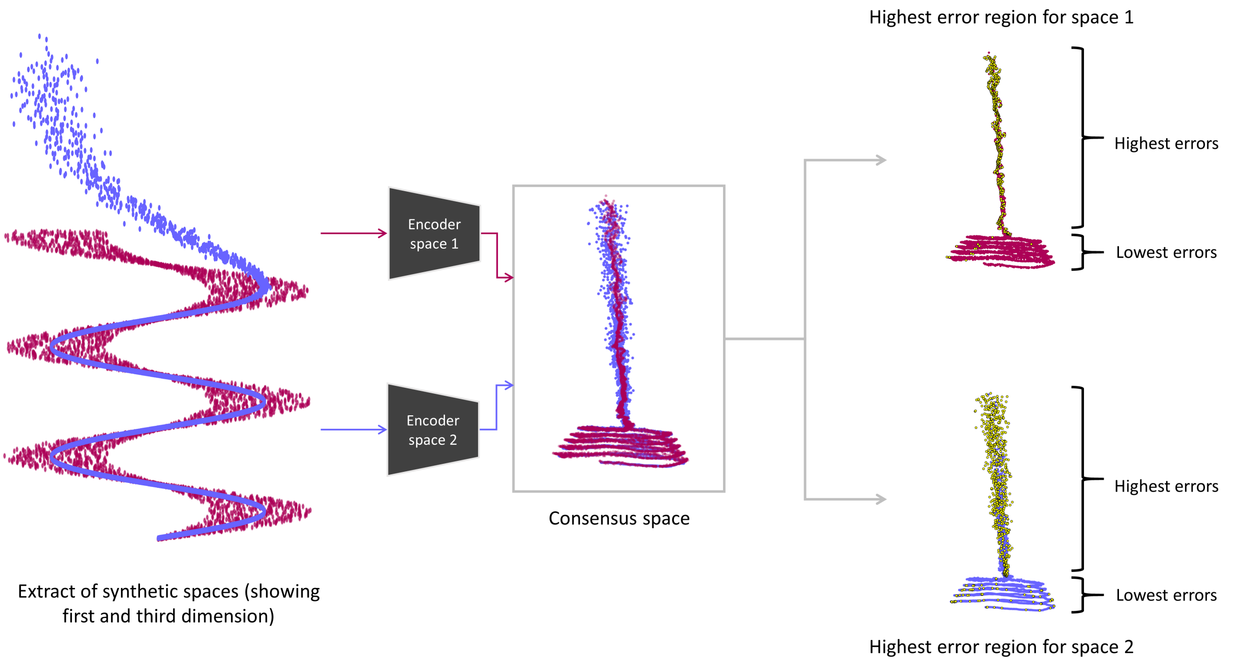

Supplementary Figure 3: FlexConsensus analysis resulting from comparing two synthetic spaces (in blue and red) with different intrinsic dimensions based on a helix curve and a helix-shaped surface. In addition, half of the turns in the helix-shaped space are stretched and corrupted with noise depending on the distance to the center of the helix. The resulting consensus space (of dimension 2) shows the capacity of the method to identify the common and uncommon regions present in the input spaces. To the right of the Figure, we present the two isolated consensus spaces obtained from the inputs, with those points estimated to have the largest consensus errors highlighted in yellow.

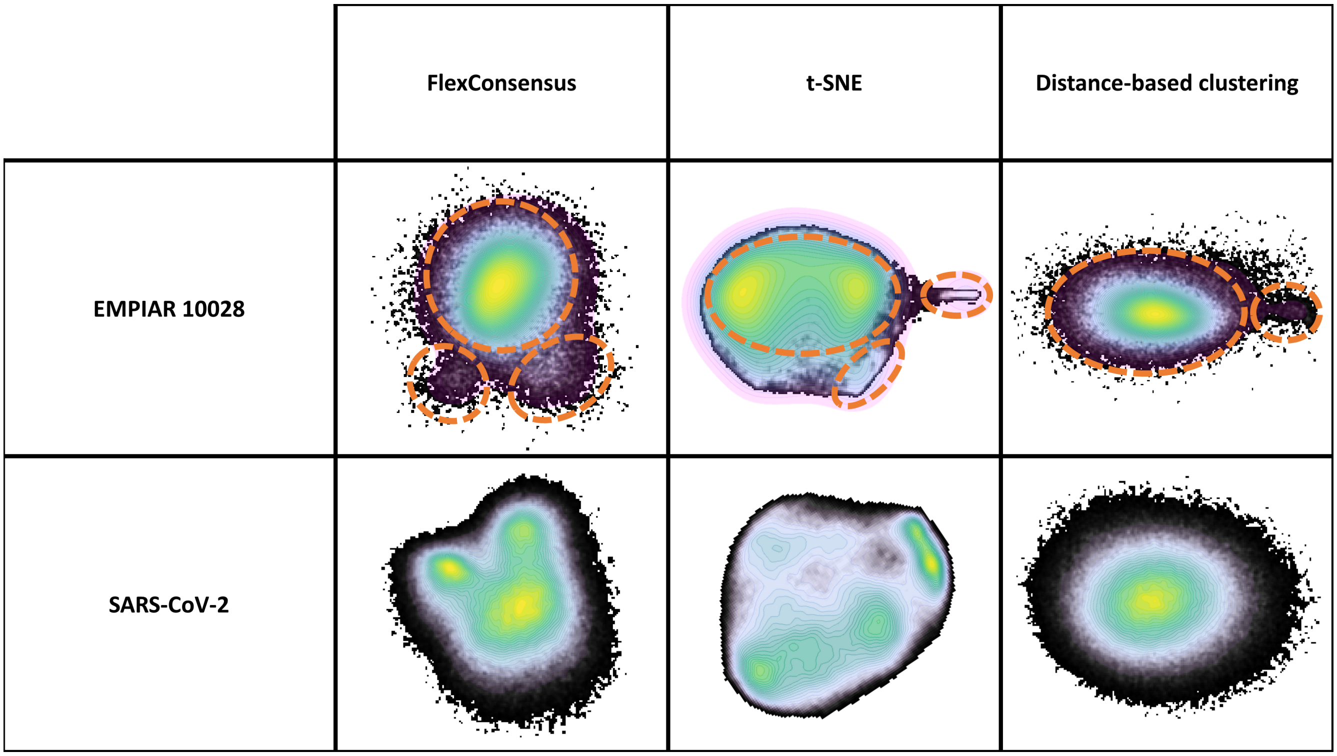

Supplementary Figure 4: Comparison of the dimensionality reduction capabilities of FlexConsensus against t-SNE and distance-based clustering. For the two datasets presented, the original HetSIREN executions analyzed in the manuscript were used as the input for each algorithm.

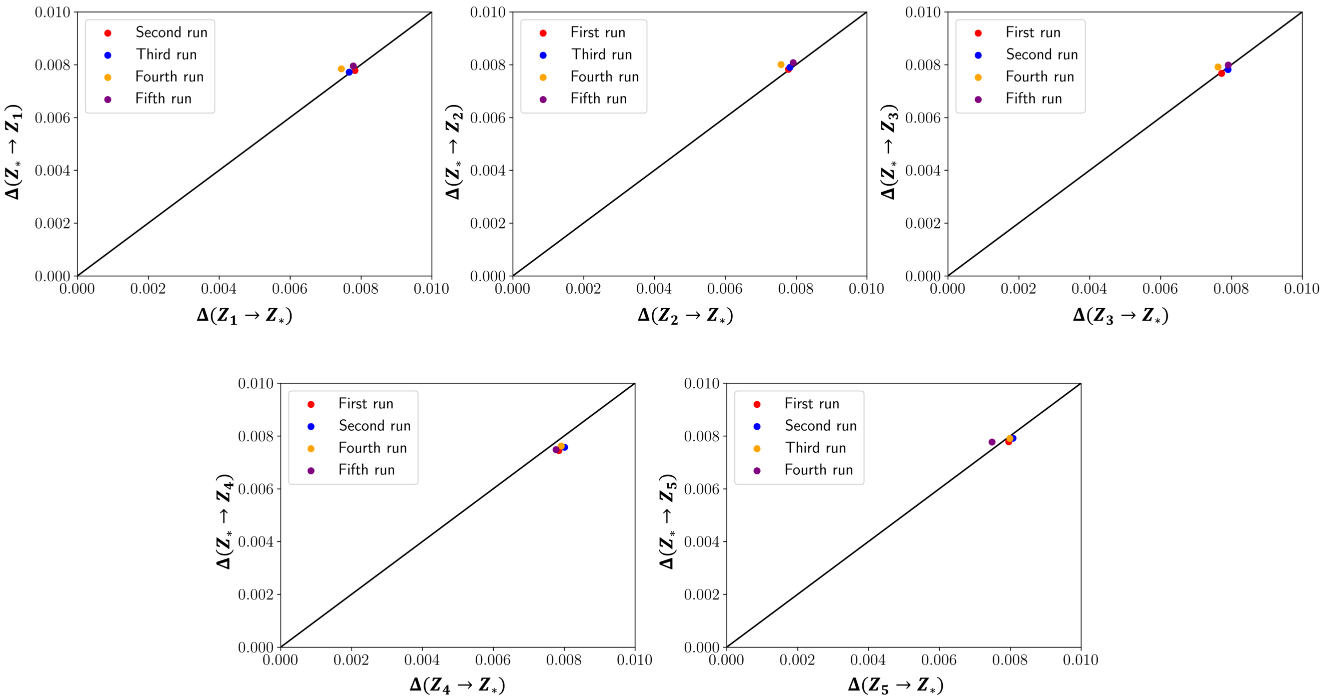

Supplementary Figure 5: Information imbalance plots obtained from independent runs of FlexConsensus trained with the helix-based synthetic dataset presented in the manuscript. The plots demonstrate that FlexConsensus executions yield similar consensus landscapes after training, indicating the reproducibility of the results.
