# Supplementary material for: Merging conformational landscapes in a single consensus space with FlexConsensus algorithm
Source: Nat Methods. 2025 Sep 25;22(10):2118–26. doi: 10.1038/s41592-025-02841-w (PMC12510870; doi:10.1038/s41592-025-02841-w)
Supplement: Supplementary file 2 — Reporting Summary [file 41592_2025_2841_MOESM2_ESM.pdf]

nature portfolio

Corresponding author(s): David Herreros

Last updated by author(s): Jun 24, 2025

Reporting Summary

Nature Portfolio wishes to improve the reproducibility of the work that we publish. This form provides structure for consistency and transparency in reporting. For further information on Nature Portfolio policies, see our Editorial Policies and the Editorial Policy Checklist.

Statistics

For all statistical analyses, confirm that the following items are present in the figure legend, table legend, main text, or Methods section.

n/a	Confirmed	
☐	☒	The exact sample size (*n*) for each experimental group/condition, given as a discrete number and unit of measurement
☒	☐	A statement on whether measurements were taken from distinct samples or whether the same sample was measured repeatedly
☐	☒	The statistical test(s) used AND whether they are one- or two-sided<br>*Only common tests should be described solely by name; describe more complex techniques in the Methods section.*
☒	☐	A description of all covariates tested
☒	☐	A description of any assumptions or corrections, such as tests of normality and adjustment for multiple comparisons
☐	☒	A full description of the statistical parameters including central tendency (e.g. means) or other basic estimates (e.g. regression coefficient) AND variation (e.g. standard deviation) or associated estimates of uncertainty (e.g. confidence intervals)
☐	☒	For null hypothesis testing, the test statistic (e.g. *F*, *t*, *r*) with confidence intervals, effect sizes, degrees of freedom and *P* value noted<br>*Give P values as exact values whenever suitable.*
☒	☐	For Bayesian analysis, information on the choice of priors and Markov chain Monte Carlo settings
☒	☐	For hierarchical and complex designs, identification of the appropriate level for tests and full reporting of outcomes
☒	☐	Estimates of effect sizes (e.g. Cohen's *d*, Pearson's *r*), indicating how they were calculated

Our web collection on statistics for biologists contains articles on many of the points above.

Software and code

Policy information about availability of computer code

Data collection	No data was collected for this manuscript.
Data analysis	All the data was analyzed with Scipion 3.8.0 software package. Inside Scipion, CryoDRGN v3.4.0 was also used to estimate the conformational landscapes analyzed in the manuscript, as well as Flexutils plugin v3.3.0.<br><br>CryoSPARC 4.5.1, Relion 4.0, and Xmipp 3.24.12.0 packages were also used to preprocess the data.<br><br>FlexConsensus algorithm is available through Scipion 3.0 under the plugins and "scipion-em-flexutils" (https://github.com/scipion-em/scipion-em-flexutils) and the package "Flexutils-Toolkit" (https://github.com/I2PC/Flexutils-Toolkit).

For manuscripts utilizing custom algorithms or software that are central to the research but not yet described in published literature, software must be made available to editors and reviewers. We strongly encourage code deposition in a community repository (e.g. GitHub). See the Nature Portfolio guidelines for submitting code & software for further information.

Data

Policy information about availability of data

All manuscripts must include a data availability statement. This statement should provide the following information, where applicable:

- Accession codes, unique identifiers, or web links for publicly available datasets
- A description of any restrictions on data availability
- For clinical datasets or third party data, please ensure that the statement adheres to our policy

The experimental datasets analyzed in the manuscript are available in EMPIAR under the entries: 10028 [https://doi.org/10.6019/EMPIAR-10028]. The e SARS-CoV-2 D614 spike dataset will be published in a different work in the future. CryoBench datasets are publicly available through the following link https://cryobench.cs.princeton.edu/

Research involving human participants, their data, or biological material

Policy information about studies with human participants or human data. See also policy information about sex, gender (identity/presentation), and sexual orientation and race, ethnicity and racism.

Reporting on sex and gender	Not applicable
Reporting on race, ethnicity, or other socially relevant groupings	Not applicable
Population characteristics	Not applicable
Recruitment	Not applicable
Ethics oversight	Not applicable

Note that full information on the approval of the study protocol must also be provided in the manuscript.

Field-specific reporting

Please select the one below that is the best fit for your research. If you are not sure, read the appropriate sections before making your selection.

- [x] Life sciences
- [ ] Behavioural & social sciences
- [ ] Ecological, evolutionary & environmental sciences

For a reference copy of the document with all sections, see nature.com/documents/nr-reporting-summary-flat.pdf

Life sciences study design

All studies must disclose on these points even when the disclosure is negative.

Sample size	This information is not relevant for image processing data.
Data exclusions	No data was excluded from the analyses.
Replication	All experiments were repeated more than once to ensure that the results and conclusions derived from them were reproducible.
Randomization	This is not relevant to this study, as the datasets are independent and cannot be mixed into groups.
Blinding	This is not relevant for this study, as the datasets are not blind during their analysis.

Reporting for specific materials, systems and methods

We require information from authors about some types of materials, experimental systems and methods used in many studies. Here, indicate whether each material, system or method listed is relevant to your study. If you are not sure if a list item applies to your research, read the appropriate section before selecting a response.

Materials & experimental systems

n/a	Involved in the study
☒	☐ Antibodies
☒	☐ Eukaryotic cell lines
☒	☐ Palaeontology and archaeology
☒	☐ Animals and other organisms
☒	☐ Clinical data
☒	☐ Dual use research of concern
☒	☐ Plants

Methods

n/a	Involved in the study
☒	☐ ChIP-seq
☒	☐ Flow cytometry
☒	☐ MRI-based neuroimaging

Plants

Seed stocks	Not applicable
Novel plant genotypes	Not applicable
Authentication	Not applicable
